# Multilocus Sequence Typing and Virulence Profiles in Uropathogenic *Escherichia coli* Isolated from Cats in the United States

**DOI:** 10.1371/journal.pone.0143335

**Published:** 2015-11-20

**Authors:** Xiaoqiang Liu, Kamoltip Thungrat, Dawn M. Boothe

**Affiliations:** 1 College of Veterinary Medicine, Northwest A&F University, Yangling, Shaanxi 712100, China; 2 Department of Anatomy, Physiology and Pharmacology, College of Veterinary Medicine, Auburn University, Auburn, Alabama 36849, United States of America; Iowa State University, UNITED STATES

## Abstract

The population structure, virulence, and antimicrobial resistance of uropathogenic *E*. *coli* (UPEC) from cats are rarely characterized. The aim of this study was to compare and characterize the UPEC isolated from cats in four geographic regions of USA in terms of their multilocus sequence typing (MLST), virulence profiles, clinical signs, antimicrobial resistance and phylogenetic grouping. The results showed that a total of 74 *E*. *coli* isolates were typed to 40 sequence types with 10 being novel. The most frequent phylogenetic group was B2 (n = 57). The most frequent sequence types were ST73 (n = 12) and ST83 (n = 6), ST73 was represented by four multidrug resistant (MDR) and eight non-multidrug resistant (SDR) isolates, and ST83 were significantly more likely to exhibit no drug resistant (NDR) isolates carrying the highest number of virulence genes. Additionally, MDR isolates were more diverse, and followed by SDR and NDR isolates in regards to the distribution of the STs. *afa/draBC* was the most prevalent among the 29 virulence-associated genes. Linking virulence profile and antimicrobial resistance, the majority of virulence-associated genes tested were more prevalent in NDR isolates, and followed by SDR and MDR isolates. Twenty (50%) MLST types in this study have previously been associated with human isolates, suggesting that these STs are potentially zoonotic. Our data enhanced the understanding of *E*. *coli* population structure and virulence association from cats. The diverse and various combinations of virulence-associated genes implied that the infection control may be challenging.

## Introduction

Urinary tract infection (UTI) is one of the most frequent bacterial infection in both human and companion animals. Uropathogenic *Escherichia coli* (UPEC), belonging to extraintestinal pathogenic *E*. *coli* (ExPEC), is the most common bacterium isolated from canine and feline UTIs [1]. Considering the physical closeness in which many humans live with their pet companions, these organisms are responsible for significant social and economic costs for both communities and public health resources as previous study indicate that there may be cross transmission of ExPEC between animals and humans [2]. The ability of UPEC to cause symptomatic UTIs is associated with expression of numerous virulence-associated genes. This includes a broad array of adhesins, invasins, toxins and proteins, which are responsible for pathogenesis outside the gastrointestinal tract [3,4]. Virulence-associated genes are present in increased numbers in UPEC compared to *E*. *coli* that remain in the gastrointestinal tract. The presence of some genes, such as *papA*, *papC*, *sfa/foc*, *afa/draBC*, *iutA* and *kpsMT* II are often responsible for acute UTIs [5,6]. Previous surveys have demonstrated similarities among clinical *E*. *coli* isolates from humans, dogs, and cats with respect to genomic background and virulence genes, suggesting possible zoonotic transmission [7].

Transmission of antimicrobial resistance among bacterial isolates is an increasing problem in infectious diseases [8]. Antimicrobial susceptible isolates mostly derive from phylogenetic group B2 and are associated with higher virulence-associated genes prevalence than antimicrobial resistant isolates, which are typically associated with shifts toward groups D and A [9]. Some studies have shown that virulence of *E*.*coli* isolates are sometimes associated with antimicrobial resistance, whereas other studies have reported that antimicrobial resistance and virulence-associated genes are only weakly linked [8,10].

Knowledge of the molecular pathogenicity of ExPEC infections has improved markedly over the last decade, however, the genetic background and virulence profiles of feline UPEC have been studied to a much lesser extent or in much smaller sample populations in the *E*. *coli* isolates from cats. The aim of the current study was to compare and characterize the UPEC isolated from cats in the United States in terms of their virulence, antimicrobial resistance profiles, phylogenetic grouping, and multilocus sequence typing (MLST) in order to obtain a comprehensive understanding of the population structure of UPEC.

## Materials and Methods

### Bacterial Isolates

We have a primary surveillance study population consisting of *740 E*. *coli* isolates acquired from cats located in four geographic regions in the United States: West (California), South (North Carolina), Midwest (Ohio and Illinois), and Northeast (Massachusetts) from January 2008 through January 2013. Organisms isolated from the urine of cats with suspected UTI had been submitted to a nationally recognized veterinary diagnostic laboratory that receives samples throughout the United States. Organisms had been isolated and identified as *E*. *coli* isolates. Upon receipt by Clinical Pharmacology Laboratory (CPL) at Auburn University, identification as *E*. *coli* was reconfirmed based on reculture overnight on BBL CHROMagar Orientation (BD Diagnostics, Franklin Lakes, NJ) at 37°C. And then, the isolates were harvested and stored in trypticase soy broth containing 30% glycerol at -80°C for further analysis.

### Antimicrobial Susceptibility Testing and Extended-spectrum β-lactamases (ESBLs) Detection

Antimicrobial susceptibility testing was performed for all isolates using custom microdilution susceptibility plates according to the manufacturer’s protocol (Trek Diagnostic Systems, Inc., Cleveland, OH). A panel of sixteen drugs was studied, representing six drug classes, classified into 12 antimicrobial categories: penicillins, penicillins + β-lactam inhibitors, antipseudomonal + β-lactam inhibitor, non-extended spectrum cephalosporins (1^st^ generation cephalosporins), extended-spectrum cephalosporins (3^rd^ and 4^th^ generation cephalosporins), cephamycins, carbapenems, tetracyclines, phenicols, fluoroquinolones, folate pathway inhibitors, and aminoglycosides was tested [11,12], the antibacterial agents and the corresponding breakpoints were listed in S1 Table), and the MICs were recorded using the Sensititre Vizion system (Trek Diagnostic Systems) (The breakpoint MICs of the drugs tested in S1 Table). All MIC determinations were performed in triplicate and reference strain *E*. *coli* ATCC 25922 was used for quality control. The results were interpreted by the guidelines of CLSI [13]. Each isolate was categorized as no drug (NDR), non-multidrug (SDR), multidrug (MDR) resistance. SDR was defined as resistance to 1 or 2 of the previously described 12 antimicrobial categories. MDR was defined as resistance to three or more categories [11].

Additionally, the *E*. *coli* isolates were tested for extended-spectrum β-lactamase (ESBL) production using microdilution-based Sensititre (Trek Diagnostic Systems, Inc., Cleveland, OH) with ESBL Confirmatory MIC plates (ESB1F) according to previous study of our laboratory [14].

Seventy four (10% of primary surveillance study population isolates, including all of NDR [n = 12], with the remaining comparised of isolates expression SDR [n = 24] and MDR [n = 36]). The SDR and MDR isolates represent 9% of isolates in the entire population expressing SDR and MDR respectively and were randomly selected. Among the 74 patients, 15 (20.3%) were male and 59 (79.7%) were female, and 58 (78.4%) of them are more than 10 years old. Additionally, each isolate was classified in terms of the severity of clinical signs as to absent, mild, moderate, severe or life-threating with the latter generally reflecting pyelonephritis with urosepsis. The scoring system reflected the clinical impression of the veterinarian in consultation with the pet owner. Further, each isolate was also designated as either absent (asymptomatic bacteriuria [ABU]) versus non-ABU (mild, moderate, severe, and life-threatening).

### Phylogenetic Grouping and Virulence Genotyping

The distribution of phylogenetic groups amongst UPEC isolates was determined as recently improved phylotyping PCR approach described by Clermont and colleagues [15]. PCR virulence typing was performed in the triplex and duplex PCR reactions as previously described [16] or conventional PCR reaction. All *E*. *coli* isolates tested were screened for 29 virulence-associated genes of extraintestinal *E*. *coli*, including a pathogenicity associated island (PAI), representing six categories: adhesins (*fimH*, *papA*, *pap*C, *papE*, *papG*, *papG* I, *papG* II, *papG* III, *sfa*/*focDE*, *afa/draBC*, *sfaS*, *focA*, *focG*, and *bmaE*), toxins (*hlyA*, *hlyD* and *cnf1*), capsule synthesis (*kpsMT* II, *kpsMT* K1, *kpsMT* K5 and *rfc*), siderophores (*fyuA*, *iroN*, *ireA* and *iutA*), invasin (*ibeA*) and miscellaneous genes (*traT*, PAI and *cvaC*). The primer sequences are listed in Table 1.

**Table 1:** The Oligonucleotide primers of virulence-associated genes used in this study.

Target gene	Accession No.	Primer sequence (5’-3’)	Fragment size (bp)	Reference
*fimH*	AJ225176	TGCAGAACGGATAAGCCGTGG/ GCAGTCACCTGCCCTCCGGTA	508	[16]
*papA*	X61239	ATGGCAGTGGTGTCTTTTGGTG/ CGTCCCACCATACGTGCTCTTC	717	[16]
*papC*	X61239	GTGGCAGTATGAGTAATGACCGTTA/ ATATCCTTTCTGCAGGGATGCAATA	205	[16]
*papE*	X61239	GCAACAGCAACGCTGGTTGCATCAT/ AGAGAGAGCCACTCTTATACGGACA	336	[16]
*papG*	X61239	CTGTAATTACGGAAGTGATTTCTG/ ACTATCCGGCTCCGGATAAACCAT	1070	[16]
*papG* I	X61239	TCGTGCTCAGGTCCGGAATTT/ TCCAGAAATAGCTCATGTAACCCG	479	[40]
*papG* II	M20181	GGGATGAGCGGGCCTTTGAT/ CGGGCCCCCAAGTAACTCG	190	[41]
*papG* III	X61238	GGCCTGCAATGGATTTACCTGG/ CCACCAAATGACCATGCCAGAC	258	[41]
*sfa/focDE*	Unpublished	CTCCGGAGAACTGGGTGCATCTTAC/CGGAGGAGTAATTACAAACCTGGCA	410	[42]
*afa/draBC*	X76688	GGCAGAGGGCCGGCAACAGGC/CCCGTAACGCGCCAGCATCTC	559	[41]
*sfaS*	S53210	GTGGATACGACGATTACTGTG/CCGCCAGCATTCCCTGTATTC	240	[16]
*focA*	DQ301498	ATGCGTCYGCTGTCACCACGG/ GGCGTCGGCGTTGGCAATAC	458	This study
*focG*	DQ301498	CAGCACAGGCAGTGGATACGA/GAATGTCGCCTGCCCATTGCT	364	[16]
*bmaE*	M15677	ATGGCGCTAACTTGCCATGCTG/ AGGGGGACATATAGCCCCCTTC	507	[16]
*hlyA*	M10133	AACAAGGATAAGCACTGTTCTGGCT/ACCATATAAGCGGTCATTCCCGTCA	1177	[43]
*hlyD*	AM690759	CTCCGGTACGTGAAAAGGAC/ GCCCTGATTACTGAAGCCTG	904	[16]
*cnfI*	U42629	ATCTTATACTGGATGGGATCATCTTGG/ GCAGAACGACGTTCTTCATAAGTAT	974	[43]
*kpsMT* II	X53819	GCGCATTTGCTGATACTGTTG/ CATCCAGACGATAAGCATGAGCA	272	[16]
*kpsMT* K1	M57382	TAGCAAACGTTCTATTGGTGC/ CATCCAGACGATAAGCATGAGCA	153	[16]
*kpsMT* K5	X53819	CAGTATCAGCAATCGTTCTGTA/ AACCATACCAACCAATGCGAG	159	[16]
*rfc*	U39042	ATCCATCAGGAGGGGACTGGA/ CATCCAGACGATAAGCATGAGCA	788	[16]
*fyuA*	Z38064	TGATTAACCCCGCGACGGGAA/ CGCAGTAGGCACGATGTTGTA	880	[16]
*iroN*	CP001671	AAGTCAAAGCAGGGGTTGCCCG/ GACGCCGACATTAAGACGCAG	667	This study
*ireA*	AF320691	GATGACTCAGCCACGGGTAA/ CCAGGACTCACCTCACGAAT	254	This study
*iutA*	X05874	GGCTGGACATCATGGGAACTGG/ CGTCGGGAACGGGTAGAATCG	302	[44]
*ibeA*	L42624	AGGCAGGTGTGCGCCGCGTAC/ TGGTGCTCCGGCAAACCATGC	170	[16]
*traT*	J01769	GGTGTGGTGCGATGAGCACAG/ CACGGTTCAGCCATCCCTGAG	290	[16]
PAI	AF003742	GGACATCCTGTTACAGCGCGCA/ TCGCCACCAATCACAGCCGAAC	930	[16]
*cvaC*	X57525	CACACACAAACGGGAGCTGTT/ CTTCCCGCAGCATAGTTCCAT	680	[16]

### Multilocus Sequence Typing

All 74 UPEC isolates were assigned to multilocus sequence types as described previously [17]. PCR amplification and sequencing of seven housekeeping genes (*adk*, *fumC*, *gyrB*, *icd*, *mdh*, *purA* and *recA*) were performed following the protocols specified at the *E*. *coli* MLST website (http://mlst.warwick.ac.uk/mlst/dbs/Ecoli). All the primer sequences of seven genes are available at http://mlst.warwick.ac.uk/mlst/dbs/Ecoli/documents/primersColi_html. The 50 μl amplification reaction mixture comprised 2 μl of template DNA, 1.5 μl of each primer (25 pmol/μl), 25 μl 2×PCR Super Master Mix (Biotool LLC., TX) and 20 μl sterilized distilled water. The reaction conditions were an initial denaturation step at 94°C for 2 min, followed by 30 cycles of the following conditions: denaturation at 94°C for 1 min, 1 min primer annealing at 54–60°C, and extension at 72°C for 2 min, with a final extension step at 72°C for 5 min.

Amplicons from seven housekeeping genes were purified using a QIAquick PCR Purification Kit (Qiagen, Inc., Valencia, CA). Sequencing of the PCR products was performed using the services of Macrogen (Macrogen Inc., Rockville, MD), and then alignment between the sequences reference sequences of *E*. *coli* MG1655 were done using MEGA version 6.0 software. Allele numbers for seven gene fragments of each isolate were obtained by comparing with corresponding allele available in MLST *E*. *coli* database (http://mlst.warwick.ac.uk/mlst/dbs/Ecoli), and Sequence type (ST) of each isolate was determined by combining seven allelic profiles.

### Statistical Analysis

Significance was determined by Pearson’s Chi-squared test with Yates continuity correction using ‘R’ software (version 3.0.1). The threshold for statistical significance was a *P* values of <0.05.

## Results

### Antimicrobial Susceptibility

The antimicrobial susceptibility results showed that 72.4% (62/74) expressed resistance to at least one antimicrobial drug, including 26 (35.1%) SDR phenotype and 36 (48.6%) MDR phenotype isolates. Only 12 (16.2%) isolates were fully susceptible to all antimicrobials tested (NDR). Regarding resistance to drugs, the highest rates of resistance was expressed toward doxycycline with100% (62/62), and followed by cephalothin at 98.4% (61/62), and ampicillin (62.9%), and the least prevalence was meropenem (4.8%). The percentages of severity were moderate (39.2%; 29/74), followed by mild (18.9%), severe (24.3%) and life-threatening (4.1%). The ABU isolates represented 13.5% (10/74) of total feline UPEC isolates.

### Phylogenetic Groups

As is present in Table 2, the predominant phylogenetic group was B2 (74.3%, 55/74), followed by B1 (9.5%, 7/74), D (6.8%, 6/74), A (2.7%, 2/74), E (2.7%, 2/74), and F (2.7%, 2/74) (*P* < 0.001, B2 *vs*. B1, D, A, E and F). Phylogenetic group B2 also accounted for 91.7%, 76.9% and 66.7% of the NDR, SDR and MDR phenotype isolates, respectively. Additionally, the isolates from groups A and E expressed MDR phenotype. Phylogenetic analysis in relation to severity of clinical signs revealed that the majority of isolates from patients were considered clinically severe, and belonged to group B2 (53.8%; 10/18). While all the group D isolates belonged to ABU isolates.

**Table 2:** The prevalence of virulence-associated genes among 74 uropathogenic *E*. *coli* isolates from cats.

	Prevalence [no. (%)] of virulence genes					*P* value	
	Total number(n = 74)	NDR (n = 12)	SDR (n = 26)	MDR (n = 36)	NDR*vs*SDR	NDR*vs*MDR	SDR*vs*MDR
**Phylogenetic group**							
B2	55 (74.3)	11 (91.7)	20 (76.9)	24 (66.7)	0.009	0.007	0.092
B1	7 (9.5)	1 (8.3)	4 (15.4)	2 (5.6)	0.001	0.008	0.054
D	6 (8.1)	0 (0)	0 (0)	6 (13.9)	1	<0.0001	<0.0001
A	2 (2.7)	0 (0)	0 (0)	2 (5.6)	1	<0.0001	<0.0001
E	2 (2.7)	0 (0)	0 (0)	2 (5.6)	1	<0.0001	<0.0001
F	2 (2.7)	0 (0)	2 (7.7)	0 (0)	<0.0001	1	<0.0001
**Virulence determinants**							
**Adhesins**							
*afa/draBC*	68 (91.9)	12 (100)	24 (92.3)	32 (88.9)	0.465	0.587	0.309
*fimH*	64 (86.5)	11 (91.7)	23 (88.5)	30 (83.3)	0.302	0.302	0.204
*papA*	47 (63.5)	11 (91.7)	16 (61.5)	20 (55.6)	0.000	0.000	0.112
*papE*	47 (63.5)	9 (75.0)	16 (61.5)	22 (61.1)	0.017	0.044	0.178
*sfa*/*focDE*	47 (63.5)	10 (83.3)	20 (76.9)	17 (47.2)	0.068	0.008	0.004
*papC*	45 (60.8)	10 (83.3)	18 (69.2)	17 (47.2)	0.038	0.008	0.018
*focG*	44 (59.5)	10 (83.3)	16 (61.5)	18 (50.0)	0.000	0.006	0.048
*papG*	21 (28.4)	9 (75.0)	11 (42.3)	1 (2.8)	0.000	0.000	0.000
*focA*	31 (41.9)	5 (41.7)	13 (50.0)	13 (36.1)	0.143	0.068	0.038
*papG* III	30 (40.5)	11 (91.7)	8 (30.8)	11 (30.6)	0.000	0.000	0.957
*sfaS*	9 (12.2)	1 (8.3)	5 (19.2)	3 (8.3)	0.002	1	0.002
*papG* II	2 (2.7)	0 (0)	2 (7.7)	0 (0)	0.086	1	0.086
*papG* I	1 (1.4)	1 (8.3)	0 (0)	0 (0)	0.092	0.092	1
*bmaE*	1 (1.4)	1 (8.3)	0 (0)	0 (0)	0.092	0.092	1
**Toxins**							
*hlyD*	56 (75.7)	11 (91.7)	19 (73.1)	26 (72.2)	0.027	0.024	0.915
*hlyA*	45 (60.8)	11 (91.7)	17 (65.4)	17 (47.2)	0.011	0.005	0.027
*cnf1*	45 (60.8)	9 (75)	18 (69.2)	18 (50.0)	0.068	0.013	0.024
**Capsule synthesis**							
*kpsMT* II	41 (55.4)	8 (66.7)	15 (57.7)	18 (50.0)	0.051	0.027	0.059
*rfc*	12 (16.2)	3 (25.0)	5 (9.2)	4 (11.1)	0.032	0.038	0.107
*kpsMTK* 5	11 (14.9)	3 (25.0)	4 (15.4)	4 (11.1)	0.051	0.038	0.077
*kpsMTK* 1	3 (4.1)	2 (16.7)	1 (3.8)	0 (0)	0.000	0.000	0.002
**Siderophores**							
*fyuA*	62 (83.8)	12 (100)	24 (92.3)	26 (72.2)	0.025	0.001	0.015
*iroN*	45 (60.8)	8 (66.7)	17 (65.4)	20 (55.6)	0.166	0.064	0.075
*iutA*	45 (60.8)	6 (50.0)	13 (50.0)	26 (72.2)	1	0.015	0.015
*ireA*	21 (28.4)	4 (33.3)	10 (38.5)	7 (19.4)	0.409	0.009	0.006
**Invasin**							
*ibeA*	13 (17.6)	4 (33.3)	4 (15.4)	5 (13.9)	0.029	0.039	0.132
**Others**							
*traT*	50 (67.6)	12 (100)	15 (57.7)	23 (63.9)	0.004	0.005	0.131
PAI	44 (59.5)	10 (83.3)	15 (57.7)	19 (52.8)	0.020	0.009	0.138
*cvaC*	6 (8.1)	4 (33.3)	0 (0)	2 (5.6)	0.000	0.000	0.128

The 29 virulence-associated genes analysed were: *afa/draBC*, Dr-binding adhesins; *fimH*, mannose-specific adhesin of type 1 fimbriae; *papA*, P fimbriae structural subunit; *papE*, fimbriae tip pilins; *papC*, p fimbriae assembly; *papG*, p fimbriae adhesin (and alleles I, II, and III); *sfa/focDE*, S and F1C fimbriae; *sfaS*, S fimbriae; *focG*, *focA*, F1C fimbriae; *bmaE*, blood group M fimbriae; *hlyD*, *hlyA*, α-haemolysin; *cnf1*, cytotoxic necrotizing factor type 1; *kpsM* II, group 2 capsule in addition to specifically targeting K1 and K5 genes; *rfc*, O antigen polymerase; *fyuA*, ferric yersiniabactin receptor; *iutA*, aerobactin receptor; *iroN*, almochelin receptor; *ireA*, iron-responsive element gene; *ibeA*, invasion of brain endothelium; *traT*, serum-resistance associated; PAI, pathogenicity island; *cvaC*, Colicin-V.

### Distribution of Virulence-associated Genes

The frequencies of the 29 virulence-associated genes were summarized in Table 2. The overall prevalence of these genes ranged from 1.4% (*papG* I and *bmaE*) to 91.9% (*afa/draBC*). The profiles of virulence-associated genes were extremely diverse, with each isolate characterized by a different profile. All of the 74 *E*. *coli* isolates harbored 2 to 24 virulence-associated genes studied. The isolates of phylogenetic groups B2 possessed averages of 14.9 virulence-associated genes, which was higher than among those in groups F, E, D, B1 and A (11.5, 10.5, 8.3, 7.7 and 4.5, respectively). Moreover, a number of virulence-associated genes such as *hlyD*, *hlyA*, *cnf1* and *iroN* were detected significantly more frequently in phylogenic group B2 compared with other groups (*P* < 0.01). While *fimH*, *sfa/focDE*, *afa/draBC*, *traT*, *iutA* and *fyuA* genes were widely distributed among all groups at different percentages.

Linking the virulence profile and clinical signs, a statistically significant difference could not be detected for the distribution of each virulence-associated genes among the five levels of severity (*P* > 0.05) with the exception of the *hlyA*, which was more prevalent in severe and life-threatening isolates. The proportion was various as isolates were categorized as either ABU *vs* non-ABU, the virulence-associated genes present in a greater proportion of non-ABU isolates were *papG* III, *focG* and *cnf1* (*P* < 0.05). The discriminator among the virulence-associated genes tested between ABU and non-ABU was *cnf1*, which occurred in 35.3% of ABU *vs* 68.4% non-ABU (*P* < 0.001). Moreover, there was a strong correlation between the distribution of the virulence-associated genes and the resistant phenotype. Overall, 100% (12/12) NDR isolates, 84.6% (22/26) SDR isolates and 61.1% (22/36) MDR isolates encoded ten or more of the 29 virulence-associated genes tested. Furthermore, the distribution rates of most genes tested were higher in NDR isolates, followed by significant descending gradients in SDR and MDR isolates with the exception of *iutA*, *sfaS*, *focA*, *papG* II and *ireA* (Table 2), the NDR, SDR and MDR phenotype isolates harbored an average of 17.4, 13.5 and 10.9 virulence-associated genes, respectively. It is noteworthy that *papG* III was significantly higher in NDR isolates (91.7%) than in SDR (30.8%) and MDR phenotype (30.6%) isolates (*P* < 0.001). Among the vast majority of the SDR or MDR phenotype isolates, resistance to more antimicrobial or antimicrobial classes possessed less virulence-associated genes. Moreover, eight ESBL positive *E*. *coli* isolates were detected in MDR phenotype, and they were distributed in phylogenetic groups B2, D and E, and the number of virulence-associated genes present in these isolates varied between 2 and 15. However, it is difficult to link the virulence profiles with phylogenetic groups among these ESBL positive isolates as the limited isolates.

### MLST Analysis and Phylogenetic Relationships of the UPEC Isolates

**Fig 1:**
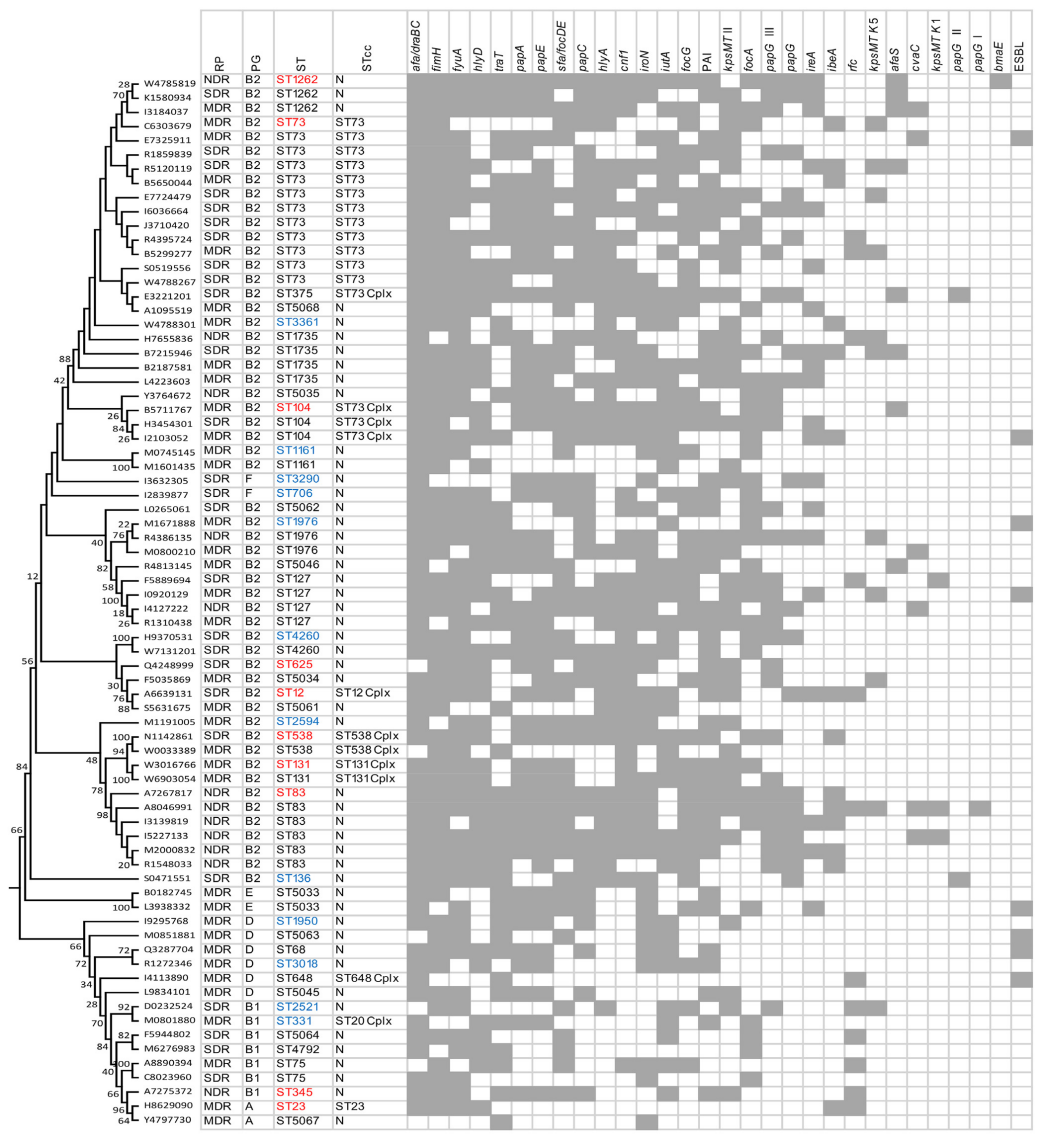
Maximum likelihood tree constructed using MEGA 6.0 based on the nucleotide sequences of seven housekeeping genes: *adk*, *gyrB*, *fumC*, *icd*, *mdh*, *purA* and *recA*, and depicting infrerred phylogency of 74 uropathogenic *E*. *coli* (UPCE) from cats. Resistant phenotype (RP), phylogenetic group (PG), sequence type (ST), ST clonal complex (STcc; “N” indicates No STcc), virulence-associated genes and the prevalence of ESBL were displayed the right of the dendrogram. Virulence-associated genes were arranged in descending order according their corresponding prevalence. Gray square indicates the presence of the virulence-associated genes and ESBL. The sequence types highlighted in red were also found to be associated with both humans and other animals, and sequence types highlighted in blue were identified in humans or animals, or in water.

We used MLST to determine the diversity and phylogenetic relationships of the UPEC isolates. Sequences were concatenated for each isolate and aligned using ClustalW in MEGA 6.0. The evolutionary history of 74 *E*. *coli* isolates tested was inferred using the maximum likelihood method based on the Tamura-Nei model and concatenated sequences of all seven genes (Fig 1). The 74 UPEC isolates analyzed were assigned to 40 distinct sequence types (STs) with ten (25%) being novel. The most frequent ST was ST73 (n = 12, 16.2%) followed by ST83 (n = 6, 8.1%), ST73 were represented by four multidrug MDR and eight SDR isolates, the ST83 isolates were significantly associated with NDR but carried the highest number of virulence-associated genes. The majority of (94.4%, 17/18) of ST73 and ST83 isolates were assigned to groups B2, and they were also associated with the severe and life-threatening situations. There were 7, 18, and 26 STs in the NDR, SDR and MDR isolates, with the Simpson's diversity index 77.3%, 91.1% and 97.9%, respectively. Two MDR isolates (2.7%) belonging to phylogenetic group B2 were ST131. Additionally, some STs or clonal complexes identified in this study have also been found to be associated with both humans and animals according to the MLST database (Fig 1, red font), and some ones were identified in humans or animals, even in water (Fig 1, blue font). Eight ESBL-producing isolates belonged to eight different STs (Fig 1), and this is the first report on ESBL positive *E*. *coli* of ST5033, ST5063 and ST104 in cats.

**Fig 2:**
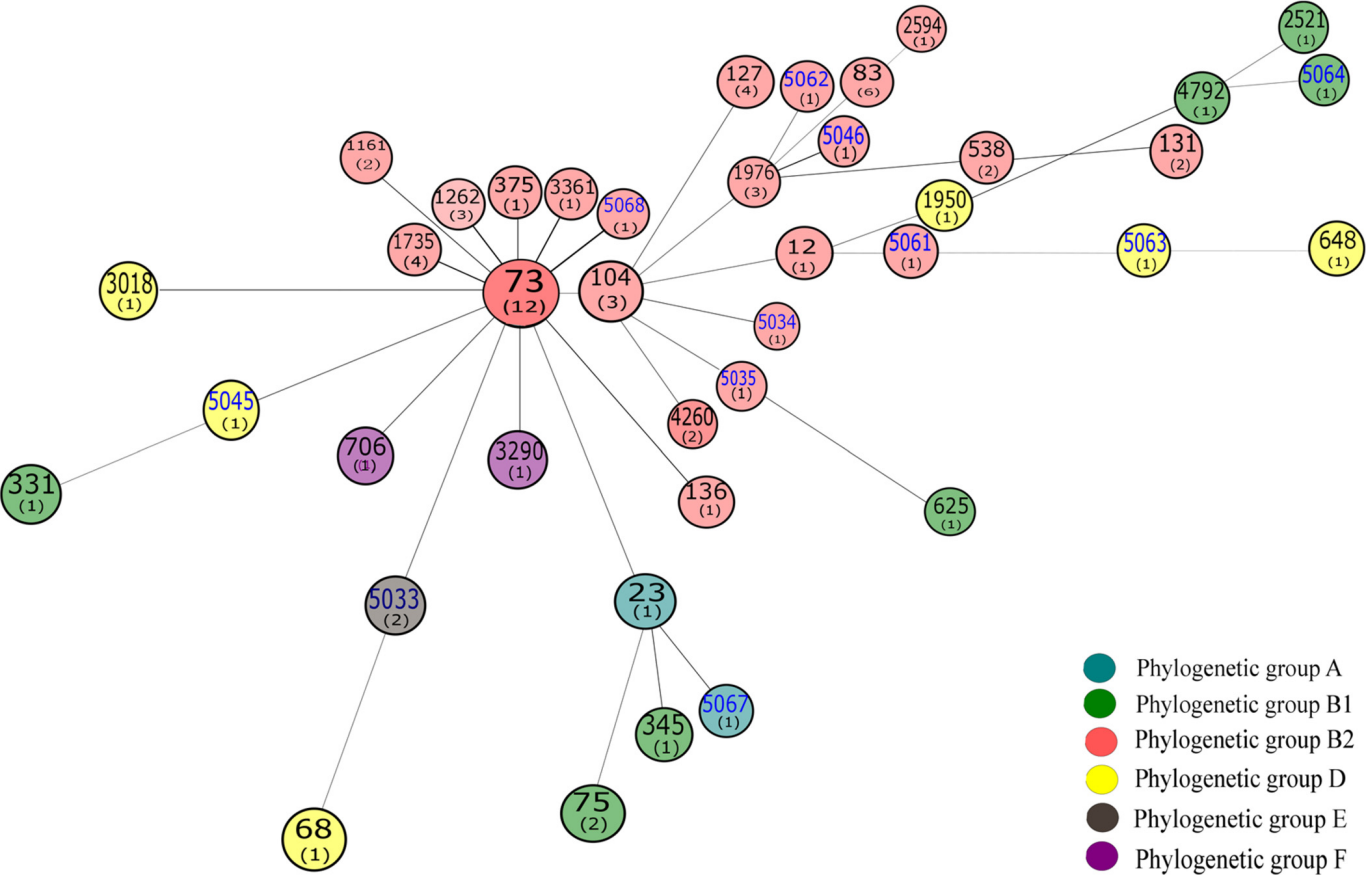
Minimum spanning (MS) tree was generated from the allelic profiles of seven housekeeping genes: *adk*, *gyrB*, *fumC*, *icd*, *mdh*, *recA* and *purA*. Each ST is represented by a circle named with its ST, and the number in the brackets of each circle represent the number of each ST in our isolates tested. The blue fonts are the novel ST identified in this study.

**Fig 3:**
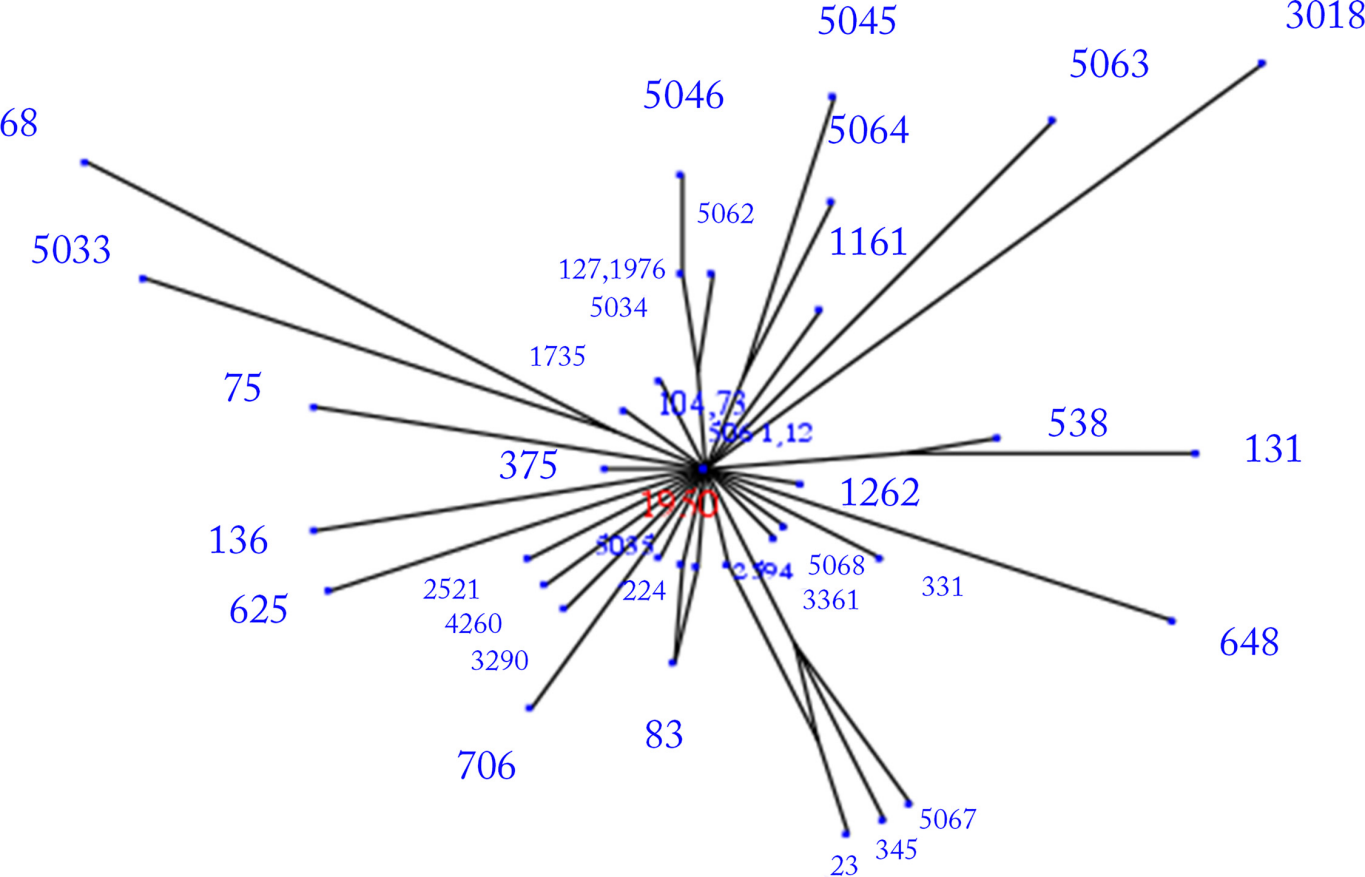
Splits tree decomposition network was obtained using distance matrix obtained from allelic profiles using a web version of Splits-Tree (http://pubmlst.org/analysis/). Most groups A and B1 isolates had shorter branches, suggesting that they were closely related as the group A and B1 isolates were considered as sister groups.

The 40 STs identified in this study were compared with all identified *E*. *coli* MLST types (In May, 2015, 7629 isolates in the *E*. *coli* MLST database belonging to 4613 STs) using the eBURST. It is clear from this analysis that these *E*. *coli* isolates were distributed widely among multiple clonal complexes (S1 Fig). In order to further evaluate the relationship between STs, the Minimum spanning (MS) tree was generated from the allelic profiles of the tested isolates using a web version of MS Tree (http://pubmlst.org/analysis/). MS tree showed that the tested *E*. *coli* mainly classed into five clonal complexes, which is represented by ST73, ST104, ST1976, ST23 and ST12, respectively. ST73 served as the predicted founder in the MS tree (Fig 2). Meanwhile, Splits tree decomposition demonstrated a similar network among *E*. *coli* STs (Fig 3). As evident from Fig 3, most group A and B1 isolates had shorter branches, suggesting that they were closely related. This is consistent with previous studies that group A and B1 isolates were considered as sister groups [18,19].

## Discussion

In the current study, we investigated the association between phylogenetic background, antimicrobial resistance, virulence profiles, clinical signs as well as the genetic relatedness of the UPEC isolated from cats in four geographic regions of the United States. Additionally, we demonstrated that UPEC from cats exhibited distinctive virulence profiles and phylogenetic background based on different clinical sign levels.

It is reported that antimicrobial susceptible and resistant ExPEC isolates are fundamentally different in bacterial populations [9,20,21]. In general, more than 94% of isolates belonged to phylogenetic groups B2, B1 and D. A similar observation has been made with ExPEC human clinical isolates [22–24]. Our data also showed that the phylogenetic profile differed between the NDR, SDR and MDR isolates. It is notable that almost all NDR isolates derived from group B2, compared with the proportion of 84.6% and 66.7%, respectively, for SDR and MDR isolates (*P* < 0.01). This was consistent with the previous study that demonstrated susceptible UPEC isolates to predominantly belong to the B2 phylogenetic group [25]. Moreover, consistently in human medicine, the proportion of group B2 is higher for severe disease, and group D was high in ABU isolates. Previous studies have found that UPEC isolates from cats share similarities with *E*. *coli* isolates that cause serious extraintestinal infections in humans. As such, *E*. *coli* also might have zoonotic and reverse zoonotic potential due to the considerable commonality observed between human and [14]animal *E*. *coli* isolates from UTIs [26,27].

Virulence profile analysis (29 genes) revealed that isolates belonging to phylogenetic groups B2 more frequently carry virulence-associated genes than that of group B1, D, A, C, E and F isolates. A high prevalence of *afa/draBC*, *fimH*, *fyuA* and *traT* (≥ 67.6%) reinforced the premise that adhesins, toxins and iron acquisition systems were more prevalent among ExPEC. Usually, fimbrial adhesins are the most common factors associated with virulent *E*. *coli* in UTI [28]. Our results firstly agreed in that *afa/draBC* (Dr-binding adhesins), a key virulence marker of ExPEC [29] was the most prevalent virulence gene. P fimbriae are the second common virulence genes of UPEC, with *pap* genes being associated with pyelonephritis [30]. In the current study, *papA*, *papC* and *papE* were present in high percentages (60.8%-63.5%), suggesting that the isolates from the urine of cats have greater capabilities to colonize kidneys and generate pyelonephritis [31]. PapG adhesin included *papG* I, *papG* II and *papG* III, and their prevalence have host specificity. According to our data, *papG* I and *papG* II were only observed in one and two isolates, respectively. It is in accordance with previous literature for which *papG* I has been observed in a lower frequency (0%–6.0%) in *E*. *coli* isolated from UTIs in cats, dogs and humans, while *papG* III was present in 95% cat isolates [32]. Our findings showed that the prevalence of *papG* III in NDR isolates (91.7%) was significantly higher prevalent than in SDR isolates (30.8%) and MDR (30.6%) isolates. These results indicated that *papG* III was the predominant *papG* allele in the pathogenesis of cat UPEC, and the isolates carrying *papG* III or not might correlate with the susceptibility or resistance of *E*. *coli*. In regards to resistance and virulence, our results revealed a strong inverse correlation between the distribution of the virulence-associated genes and the resistant phenotype. The distribution of virulence-associated genes statistically differed between and within different resistant phenotype. The vast majority of the genes tested were significantly more common in NDR isolates, followed by SDR and MDR isolates. Moreover, the prevalence of virulence-associated genes is inversely associated with antibiotic resistance, videlicet, the prevalence of virulence-associated genes was highest for NDR isolates, followed by significant descending gradients to SDR, and then MDR isolates. The exceptions were *iutA*, *sfaS*, *focA*, *papG* II and *ireA*. *iutA* was universally detected at a higher frequency in MDR isolates, and four other genes were more prevalent in SDR isolates. Meanwhile, within the majority of SDR or MDR phenotype isolates, resistance to more antimicrobial or antimicrobial categories harbored less virulence-associated genes. These findings further reinforced the previous studies that MDR *E*. *coli* from urinary tract infections tend to be associated with a decrease in the presence of virulence compared with the susceptible isolates [25,33].

The MLST was developed as a scalable typing system to determine the diversity and phylogenetic relationships of the isolates based on seven housekeeping genes, and it provides reproducibility, comparability, and transferability between laboratories [34]. Previous studies identified four major ST complexes, ST14, ST69, ST73 and ST95, associated with UPEC [35,36]. We found a highly diverse population representing 40 ST, with 10 being novel, clustered into ST73, ST104, ST1976, ST23 and ST12 clonal complexes, and 75% of STs were firstly reported in *E*. *coli* isolates from cats. The MDR phenotype isolates had a richer ST diversity (26 STs) than the SDR counterparts (18 STs) and NDR (7 STs), and MDR isolates also showed higher genetic diversity than SDR and NDR isolates with the corresponding ratio of the MLST types to the isolate number in MDR, SDR and NDR phenotype were 72.2% (26/36), 69.2% (18/26) and 58.3% (7/12). Whether or not it may imply that the genetic diversity of UPEC will gradually increase as the resistant phenotypes change from NDR, to SDR, to MDR must be further elucidated in the future studies. An interesting finding was that ST83 occurred in 50% (6/12) fully susceptible isolates, which possessed averages of 18.7 virulence-associated genes. However, it was not found in any resistant isolate. Thus far, there are four ST83 voluntarily submitted to the publicly accessible *E*. *coli* MLST database, including isolates from a cat, horse and Celebese ape. This is the first report of a relatively high prevalence of ST83 found in fully susceptible UPEC from cats. The relationship between the ST83 and resistance of *E*. *coli* needs further investigation in the future. It is worthy to mention that twenty (50%) MLST types showed relatedness to STs commonly associated with bacteremia in human patients, which provides further evidence that UPEC from cats and humans are shared.

Notably, ST131 were identified in two (2.7%) non-ESBL-producing MDR isolates associated with severe clinical sign level. However, neither isolate had a high virulence gene content nor serious resistance compared with other isolates. Previous studies have demonstrated that most ST131 are strongly associated with CTX-M-15 ESBL-producing, high virulence and fluoroquinolone resistance [37,38]. It is suggested that the isolates studied here are not a part of the pandemic clade. Nonetheless, ongoing surveillance for ST131 isolates is necessary as it is now rapidly and globally disseminated. The role in companion animals as a source or as a site of reverse zoonosis in ST131 transmission dynamics is not clear, as most companion animal ST131 isolates showed a high degree of relatedness to human ST131 isolates [39].

## Conclusion

Taken together, our results discovered a clear association between the phylogenetic groups, resistant phenotypes, virulence profiles, MLST types and different levels of clinical signs. Generally, the resistant isolates harbored less virulence-associated genes than susceptible ones, and phylogenetic groups B2 and D isolates were associated more frequently with virulence-associated genes than those belonging to other groups. MLST analysis revealed a highly diverse population representing 40 STs including 10 novel STs and two ST131 isolates, and twenty (50%) MLST types showed relatedness to STs commonly associated with humans. The most frequent MLST types in susceptible and resistant isolates were ST83 and ST73, respectively, and the genetic diversities statistically significant ascending gradients from NDR, through SDR, to MDR. Additionally, the UPEC isolates also exhibited distinctive patterns of association with virulence profiles and phylogenetic background based on different levels of clinical signs. The results significantly advance our understanding of the distinctive UPEC in feline, and may help for controlling the UTIs in future.

## Supporting Information

S1 Fig eBURST output for isolates in the entire *E*. *coli* MLST database with STs containing ExPEC isolates studied in this study ringed in pink. Blue nodes represent predicted founder STs and sub-founders are indicated in yellow, and all other STs marked as black dots.
(DOCX)

S1 Table The antimicrobial agents used in this study. (DOCX)
