# Supplementary material for: Multilocus Sequence Typing and Virulence Profiles in Uropathogenic Escherichia coli Isolated from Cats in the United States
Source: PLoS One. 2015 Nov 20;10(11):e0143335. doi: 10.1371/journal.pone.0143335 (PMC4654559; doi:10.1371/journal.pone.0143335)
Supplement: S1 Fig — Blue nodes represent predicted founder STs and sub-founders are indicated in yellow, and all other STs marked as black dots. (DOCX) [file pone.0143335.s001.docx]

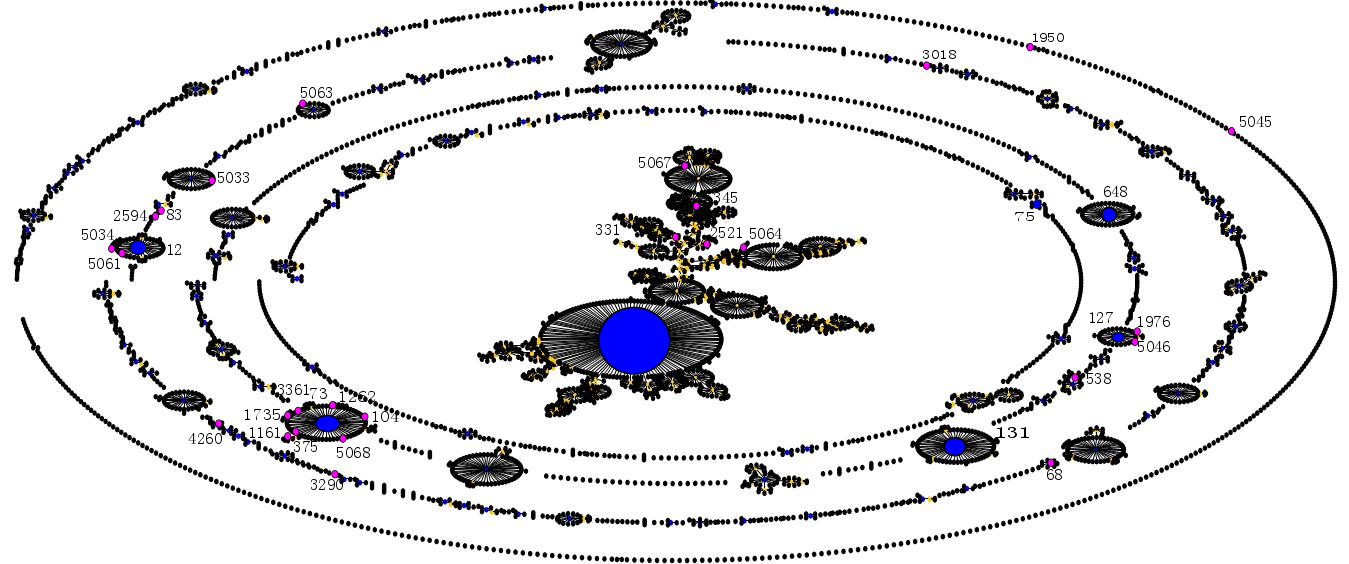


**Fig S1. eBURST output for isolates in the entire *E. coli* MLST database** with STs containing ExPEC isolates studied in this study ringed in pink. Blue nodes represent predicted founder STs and sub-founders are indicated in yellow, and all other STs marked as black dots. (Supplementary material)
