# Supplementary material for: Multilocus Sequence Typing and Virulence Profiles in Uropathogenic Escherichia coli Isolated from Cats in the United States
Source: PLoS One. 2015 Nov 20;10(11):e0143335. doi: 10.1371/journal.pone.0143335 (PMC4654559; doi:10.1371/journal.pone.0143335)
Supplement: S1 Table — (DOCX) [file pone.0143335.s002.docx]

| Table S1 The antimicrobial agents used in this study | | | |
| --- | --- | --- | --- |
|  |  |  | |
| **Antimicrobial categories** | **Conc. range tested** | **Breakpoint MIC (µg/ml)** | |
|  | **(µg/ml)** | **Susceptible** | **Resistance** |
| Penicillins |  |  |  |
| ampicillin | 0.25-256 | ≤8 ^b^ | ≥32 |
| Penicillins+β-lacatamase |  |  |  |
| amoxicillin-clavulanic acid | 0.12-1024 | ≤8/4 ^b^ | ≥32/16 |
| Antipseudomonal + β-lacatamase inhibitors |  |  |  |
| ticarcillin-clavulanic acid ^a^ | 0.25-512 | ≤16/2 | ≤128/2 |
| Non-extended spectrum cephalosporins (1st and 2nd generation cephalosporins) | | | |
| cephalothin | 0.5-1024 | ≤2 | n/a |
| Extended-spectrum cephalosporins (3rd and 4th generation cephalosporins) | | |  |
| ceftazidime ^a^ | 0.06-128 | ≤4 | ≥16 |
| cefpodoxime | 0.06-256 | ≤2 | ≥8 |
| cefotaxime | 0.06-64 | ≤1 | ≥64 |
| Cephamycin |  |  |  |
| cefoxitin ^a^ | 0.5-1024 | ≤8 | ≥32 |
| Carbapenem |  |  |  |
| meropenem | 0.06-15 | ≤1 | ≥4 |
| Tetracyclines |  |  |  |
| doxycycline | 0.25-128 | ≤0.12 | ≥0.5 |
| Phenicols |  |  |  |
| chloramphenicol | 0.5-512 | ≤8 | ≥32 |
| Fluoroquinolones |  |  |  |
| enrofloxacin | 0.008-64 | ≤0.4 | ≥4 |
| ciprofloxacin ^a^ | 0.008-128 | ≤1 | ≥4 |
| Aminoglycosides |  |  |  |
| gentamicin | 0.25-128 | ≤2 | ≥8 |
| amikacin | 0.12-128 | ≤16 | ≥32 |
| sulfonamides |  |  |  |
| trimethoprim–sulfamethoxazole ^a^ | 0.015-128 | ≤2 | ≥8 |

a Breakpoints were not veterinary-specific followed to human approved CLSI.

b Breakpoint MICs were valid for urinary tract infections only.
